# Hypoxia‐Induced ADAM23 Drives Neuron–Tumor Crosstalk and Therapeutic Resistance in Hepatocellular Carcinoma

**DOI:** 10.1002/mc.70174

**Published:** 2026-09-03

**Authors:** Taehyun Park, Soeun Kim, Hyunjung Cho, Johyeon Nam, Dayoung Choi, Misu Lee

**Affiliations:** ^1^ Division of Life Sciences, College of Life Science and Bioengineering Incheon National University Incheon Republic of Korea; ^2^ Institute for New Drug Development, College of Life Science and Bioengineering Incheon National University Incheon Republic of Korea

**Keywords:** ADAM23, hepatocellular carcinoma, HIF1α, hypoxia, neuron–tumor communication

## Abstract

Hypoxia and nutrient deprivation are fundamental drivers of tumor aggressiveness and therapeutic resistance in hepatocellular carcinoma (HCC). While the involvement of neural components in the tumor microenvironment (TME) is increasingly recognized, the molecular transducers linking metabolic stress to neuron–tumor crosstalk remain elusive. Here, we identify ADAM23 (A disintegrin and metalloproteinase 23) as a hypoxia‐responsive mediator that mediates communication between HCC cells and neuronal cells. ADAM23 expression was markedly upregulated in HCC cells under both chemical (CoCl_2_) and physical hypoxia (1% O_2_), a process further amplified by glucose deprivation and directly modulated by HIF‐1α. Functional assays revealed that ADAM23 overexpression promotes epithelial–mesenchymal transition (EMT) and enhances cell viability under metabolic stress. Notably, sorafenib‐resistant HCC cells (Huh7SR) exhibited high levels of ADAM23 secretion, which triggered proliferative and metabolic activation in neuronal SH‐SY5Y cells. In 3D co‐culture spheroid models, Huh7SR cells mixed with SH‐SY5Y cells displayed significantly larger spheroid volumes and enhanced neuronal fluorescence compared with parental controls, suggesting that ADAM23‐mediated interactions facilitate a supportive neural niche. Analysis of The Cancer Genome Atlas (TCGA) datasets and patient microarrays confirmed that ADAM23 is significantly overexpressed in HCC and positively correlates with HIF‐1α expression. Moreover, elevated expression of ADAM23 was significantly correlated with poor overall survival. Collectively, our findings underscore ADAM23 as a critical metabolic‐neural linker that promotes HCC progression and drug resistance. These findings suggest that the ADAM23‐mediated neuron–tumor axis may represent a potential therapeutic target in aggressive HCC.

## 1 Introduction

Hepatocellular carcinoma (HCC) is the predominant primary liver malignancy and remains a leading cause of cancer‐related mortality worldwide [1, 2]. Its global incidence continues to rise, driven primarily by chronic hepatitis B and C virus infections, alcohol‐associated liver disease, and the growing prevalence of non‐alcoholic fatty liver disease [2, 3]. Despite advances in diagnostic and therapeutic strategies, the prognosis for patients with advanced HCC remains poor, with a low 5‐year survival rate [4]. Tumor aggressiveness and therapeutic failure in HCC are driven by multifactorial processes, including hypoxia‐induced signaling, metabolic reprogramming—particularly alterations in glucose metabolism—and the development of chemoresistance.

Hypoxia is a critical driver of tumor progression and aggressiveness [5, 6]. Under hypoxic conditions, tumor cells undergo metabolic and transcriptional reprogramming that promotes angiogenesis, immune evasion, and resistance to anticancer therapies [7, 8, 9]. Central to these adaptive responses are hypoxia‐inducible factors (HIFs), which regulate transcriptional programs that facilitate epithelial–mesenchymal transition (EMT), cancer stem cell maintenance, and invasive behavior. In addition, nutrient limitation further complicates tumor adaptation.

Therapeutic resistance is a major determinant of poor clinical outcomes in HCC. The molecular heterogeneity of HCC and its intrinsic resistance to systemic therapies underscore the urgent need to elucidate the mechanisms underlying tumor progression and treatment failure [4, 10]. Beyond tumor heterogeneity, microenvironmental stresses—including hypoxia and nutrient deprivation—have been shown to enhance resistance to anticancer therapies across multiple tumor types. Indeed, inhibition of HIF‐1α abolishes hypoxia‐associated drug resistance, highlighting HIF‐1 activity as a critical mediator of hypoxia‐driven chemoresistance [7, 11, 12].

Accumulating evidence suggests that neural components contribute to tumor progression within the tumor microenvironment [13]. However, the roles of neuronal cells and neuron‐associated molecules in HCC remain poorly understood. Members of the ADAM (a disintegrin and metalloproteinase) family, which regulate cell adhesion, migration, and intercellular signaling, are well positioned to mediate such interactions [14, 15]. These members share a common domain structure but are encoded by distinct genes and differ substantially in catalytic activity and function; unlike the proteolytically active members, ADAM23 is catalytically inactive and acts mainly through its disintegrin and cysteine‐rich domains [15]. Notably, several ADAM members are regulated under hypoxia in liver malignancies; for example, ADAM32 is up‐regulated under hypoxic conditions in hepatoblastoma cells [16].

Among them, ADAM23 has been implicated in neuronal development, influencing neural cell growth and differentiation [15, 17]. Recent studies have also linked ADAM23 to tumorigenesis [18, 19]. In several malignancies, including breast, lung, and brain cancers, ADAM23 has been reported to function as a tumor suppressor. However, its expression pattern and biological role in HCC remain largely unexplored, and its relevance to disease progression is unclear [20]. Therefore, we aimed to elucidate the functional significance of ADAM23 in hepatocellular carcinoma, with particular emphasis on its regulation and biological effects under hypoxic and drug‐resistant conditions that characterize aggressive tumors.

## 2 Materials and Methods

### 2.1 Chemicals

Sorafenib (Santa Cruz Biotechnology, Dallas, TX, USA) was dissolved in dimethyl sulfoxide (DMSO; Sigma‐Aldrich, St. Louis, MO, USA) to prepare a 10 mM stock solution and stored at −80°C. Cobalt chloride (CoCl_2_; Sigma‐Aldrich, St. Louis, MO, USA) was dissolved in distilled water to a final concentration of 1 mM and kept at −20°C until use.

### 2.2 Cell Culture

Human hepatocellular carcinoma (HCC) cell lines (HepG2 and Huh7) and human neuroblastoma cell line SH‐SY5Y were obtained from the Korean Cell Line Bank (Seoul, Korea). Huh7 and SH‐SY5Y cells were cultured in Dulbecco's modified Eagle's medium (DMEM; Gibco, Thermo Fisher Scientific, Wilmington, DE, USA), whereas HepG2 cells were maintained in RPMI‐1640 (Thermo Fisher Scientific, Wilmington, DE, USA). All media were supplemented with 10% heat‐inactivated fetal bovine serum (FBS; HyClone, Marlborough, MA, USA) and 1% penicillin–streptomycin (Gibco). Cells were incubated at 37°C in a humidified atmosphere containing 5% CO_2_. For three‐dimensional (3D) spheroid formation, cells were seeded at 2 × 10^4^ cells/mL into ultra‐low attachment 96‐well plates (Corning, Darmstadt, Germany) and centrifuged at 179 × g for 1 min to promote single spheroid formation per well. Spheroid morphology, size, and fluorescence were monitored using a fluorescence microscope. To establish hypoxic conditions, cells were incubated in a hypoxia chamber (STEMCELL Technologies, Vancouver, Canada) flushed with a gas mixture of 1% O_2_, 5% CO_2_, and 94% N_2_ and maintained under these conditions for 24 h. Chemical hypoxia was alternatively induced by exposing cells to cobalt chloride (CoCl_2_) at concentrations ranging from 37.5 to 150 µM for 24 h.

### 2.3 Plasmid Transfection and Lentiviral Production

For overexpression of ADAM23 or HIF1A, Huh7 and Huh7R cells (1.5 × 10^5^ cells/well) and HepG2 cells (2 × 10^5^ cells/well) were seeded in six‐well plates and transfected 24 h later with 1 µg of plasmid of pCMV‐ADAM23 (Sino Biological, Eschborn, Germany), pCMV‐HIF1a (Sino Biological), or an empty control vector using FuGENE HD Transfection Reagent (Promega, Madison, WI, USA). SH‐SY5Y cells (6 × 10^5^ cells per well) were transfected with 2.5 µg of ADAM23 or HIF1A plasmid using Lipofectamine 3000 (Invitrogen, Carlsbad, CA, USA) according to the manufacturer's instructions. To generate stable GFP‐expressing SH‐SY5Y cells, lentiviral particles were produced by co‐transfecting Lenti‐X HEK293T cells (Clontech, Palo Alto, CA, USA) with the packaging plasmid psPAX2 (Addgene, Watertown, MA, USA), the envelope plasmid pCMV‐VSV‐G (Addgene, Watertown, MA, USA), and the transfer vector pLVX‐ZsGreen1‐Puro (Takara Bio Inc., Shiga, Japan). Supernatants were harvested and concentrated using the Lenti‐X Concentrator (Takara Bio Inc., Shiga, Japan) according to the manufacturer's protocol. SH‐SY5Y cells (6 × 10^5^ cells/well) were seeded in six‐well plates and infected 24 h later with the prepared viral particles diluted in medium containing 2% heat‐inactivated fetal bovine serum and 1% penicillin–streptomycin. Forty‐eight hours after infection, transduced cells were selected with 0.5 μg/mL puromycin (InvivoGen, San Diego, CA, USA) to establish a stable GFP‐positive SH‐SY5Y cell line.

### 2.4 Cell Viability Assay

Cells were seeded in 96‐well plates at 5 × 10^3^ cells/well and allowed to adhere for 24 h until ~70% confluence. Following transfection as described above, cell viability was measured after 48 h using the Cell Counting Kit‐8 (CCK‐8; Dojindo Laboratories, Kumamoto, Japan). CCK‐8 reagent (10% v/v) was added to each well and incubated according to the manufacturer's instructions. Absorbance was read at 450 nm using a SpectraMax190 microplate reader (Molecular Devices, Sunnyvale, CA, USA).

### 2.5 Quantitative Real‐Time PCR (qRT–PCR)

Total RNA was extracted using the RNeasy Mini Kit (Qiagen, Hilden, Germany) and quantified with a NanoDrop spectrophotometer (DeNovix, Wilmington, DE, USA). Complementary DNA (cDNA) was synthesized from 10 to 20 ng of total RNA using the miRCURY LNA RT Kit (Qiagen) according to the manufacturer's protocol. qRT–PCR was performed using a C1000 Thermal Cycler (Bio‐Rad Laboratories, Hercules, CA, USA) with the miRCURY LNA SYBR Green PCR Kit (Qiagen). The expression levels were normalized to housekeeping controls and analyzed using the ^ΔΔ^Ct method. The following primers were used for this assay: ADAM23 (forward, 5′‐AAGACCAGAGACAACCAGTGT‐3′ and reverse, 5′‐ACATCATGTTTGCTGCACTGA‐3′), and hGAPDH (forward, 5′‐ACAGTCAGCCGCATCTTCTT‐3′ and reverse 5′‐TTGATTTTGGAGGGATCTCG‐3′).

### 2.6 Western Blot Analysis

Cells were lysed in 1% sodium dodecyl sulfate (SDS) buffer containing 60 mM Tris–HCl and supplemented with protease (Roche, Basel, Switzerland) and phosphatase (GenDEPOT, Barker, TX, USA) inhibitors. Protein concentrations were measured using a NanoDrop spectrophotometer (DeNovix). Equal amounts of protein were loaded onto SDS–PAGE gels and transferred to PVDF membranes. After blocking in 5% skim milk, membranes were incubated overnight at 4°C with primary antibodies, followed by HRP‐conjugated secondary antibodies. Primary antibodies used in the present study were anti‐ADAM23 (LSBio, Newark, California, USA, catalog no. LS‐c680120; 1:2000), anti‐Hif1 α (Cell Signaling Technology, Danvers, MA, USA, catalog no. 36169 s; 1:1000), anti‐HKII (Abcam, Cambridge, UK, catalog no. ab209847; 1:1000), anti‐Ki67 (Dako, Copenhagen, Denmark, catalog no. M7240; 1:500), anti‐E‐cadherin (ABclonal, Woburn, MA, USA, catalog no. A20798; 1:1000), anti‐SNAIL/SLUG (Abcam, Cambridge, UK, catalog no. ab180714; 1:1000), anti‐Vimentin (Santa Cruz Biotechnology, Dallas, TX, USA, catalog no. sc‐373717; 115000), and anti‐β‐actin (Santa Cruz Biotechnology, Dallas, TX, USA, catalog no. sc‐47778; 1:5000). Protein bands were visualized using the ChemiDoc XRS detection system (Bio‐Rad), and the intensities of the protein bands were quantified using Image Lab (Bio‐Rad) and ImageJ (NIH, Bethesda, MD, USA).

### 2.7 Immunohistochemistry

A commercial tissue microarray containing 24 HCC cases (TissueArray.Com LLC, Derwood, MD, USA; catalog no. LV246) was used for immunohistochemical analysis. Staining was carried out using the ImmPRESS Duet Double Staining Polymer Kit (Vector Laboratories), enabling dual visualization with HRP (brown) and AP (magenta) chromogens. After antigen retrieval, tissues were incubated with primary antibodies against ADAM23 (LSBio; catalog no. LS‐C680120; 1:400) and HIF1α (Novus Biologicals; catalog no. NB100‐296; 1:500). Images were captured using an Olympus BX53 microscope and analyzed with CellSens software (Olympus, Tokyo, Japan).

### 2.8 Gene Expression and Survival Analyses

RSEM expected RNA counts for the The Cancer Genome Atlas (TCGA) and Genotype‐Tissue Expression (GTEx) datasets were obtained from the UCSC Xena Hub [21]. For the TCGA cohort, only patients with primary tumors and available clinical information were included. Genes were filtered based on expression abundance, retaining those with raw counts ≥ 10 in at least three samples. Differential expression analysis was performed using the R package DESeq2 [22]. Genes with a false discovery rate (FDR) < 0.05 and an absolute log2 fold change ≥ 1 were considered differentially expressed genes. Data visualization was conducted using the R package ggplot2 [23]. For survival analysis, the R packages survival and survminer were used to evaluate differences between high‐ and low‐expression groups [24, 25]. DESeq2‐normalized counts were used as input. Variance stabilizing transformation (VST) normalized counts were obtained using DESeq2 and used as input for survival and correlation analyses. For correlation analysis, Pearson correlation coefficients and associated *p*‐values were calculated using the cor.test function. Correlations with *p* < 0.05 were considered statistically significant. For survival analysis, the optimal cut‐off point for gene expression was determined using the surv_cutpoint and surv_categorize functions implemented in the survminer package. Kaplan–Meier survival curves were generated using survfit and visualized with ggsurvplot to compare survival probabilities between patient groups. A Cox proportional hazards model was applied to estimate the hazard ratio (HR) and corresponding *p*‐values.

### 2.9 Statistical Analysis

All statistical analyses were performed using GraphPad Prism 9 (GraphPad Software, San Diego, CA, USA). Comparisons between two groups were analyzed using unpaired two‐tailed Student's *t*‐tests, while multiple group comparisons were performed using one‐way ANOVA followed by Tukey's post hoc test. Correlations were determined by Pearson's correlation coefficient. Data are expressed as mean ± SEM, and *p* < 0.05 was considered statistically significant.

## 3 Results

### 3.1 Upregulation of ADAM23 Under Tumor Microenvironmental Stress in Hepatocellular Carcinoma

To investigate the expression of ADAM23 in HCC cells under hypoxic conditions, HepG2 and Huh7 cells were treated with CoCl_2_ or cultured under hypoxia (1% O_2_). The expression of ADAM23 was upregulated at both the mRNA and protein levels following CoCl_2_ treatment (Figure 1A,B). In parallel, glycolytic markers associated with tumor aggressiveness were also increased (Figure 1B–E). Consistent with these findings, exposure to 1% O_2_ similarly enhanced the expression of ADAM23 and HK2 (Figure 1F–I). To further explore the regulatory mechanism, we examined whether HIF1α, a master transcriptional regulator of the hypoxic response, modulates ADAM23 expression. Overexpression of HIF1α in HepG2 led to an increase in ADAM23 expression (Figure 1J–L). Comparable results were observed in Huh7 cells, indicating that hypoxic stress induces ADAM23 expression at both the transcriptional and translational levels (Figure 2A–L).

**Figure 1:**
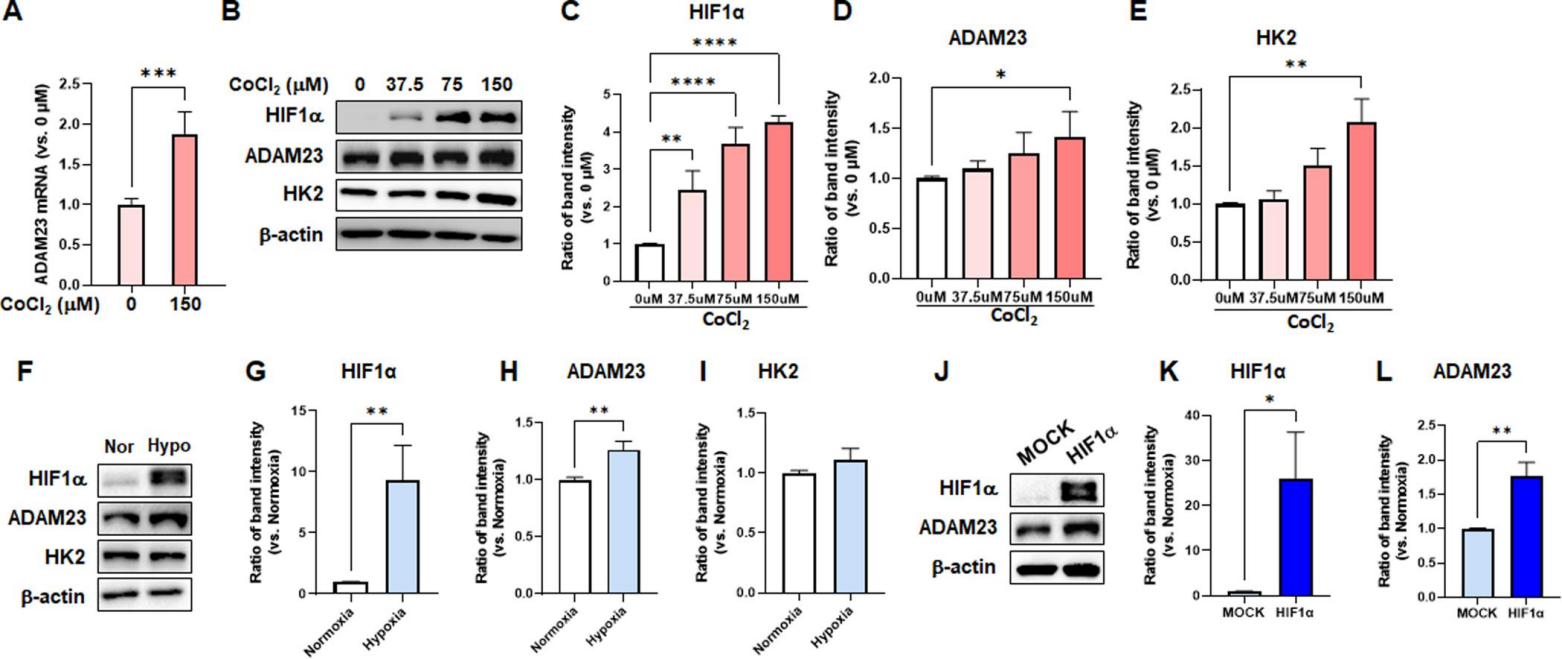
Upregulation of ADAM23 under hypoxic stress in HepG2 cells. (A) Quantitative RT–PCR analysis of ADAM23 mRNA expression in HepG2 cells treated with CoCl_2_ (150 µM, 24 h). (B) Immunoblot analysis of HIF1α, ADAM23, and HK2 in HepG2 cells following CoCl_2_ treatment (150 µM, 24 h); β‐actin served as a loading control. Full‐length blots are provided in Supporting Information S1: Figure 2A. (C–E) Densitometry intensity ratios for Figure 1B from replicated WB (*n* = 3). (F) Immunoblot analysis of HIF1α, ADAM23, and HK2 in HepG2 cells cultured under normoxic (21% O_2_) or hypoxic (1% O_2_) conditions for 24 h. Full‐length blots are provided in Supporting Information S1: Figure 3A. (G–I) Densitometry intensity ratios for Figure 1F from replicated WB (*n* = 3). (J) Immunoblot analysis of HIF1α, ADAM23, and HK2 in HepG2 cells following HIF1α overexpression. Full‐length blots in Supporting Information S1: Figure 4A. (K, L) Densitometry intensity ratios for Figure 1J from replicated WB (*n* = 3). Data are presented as mean ± SEM from three independent experiments. *p < 0.05, **p < 0.01, ****p < 0.0001.

**Figure 2:**
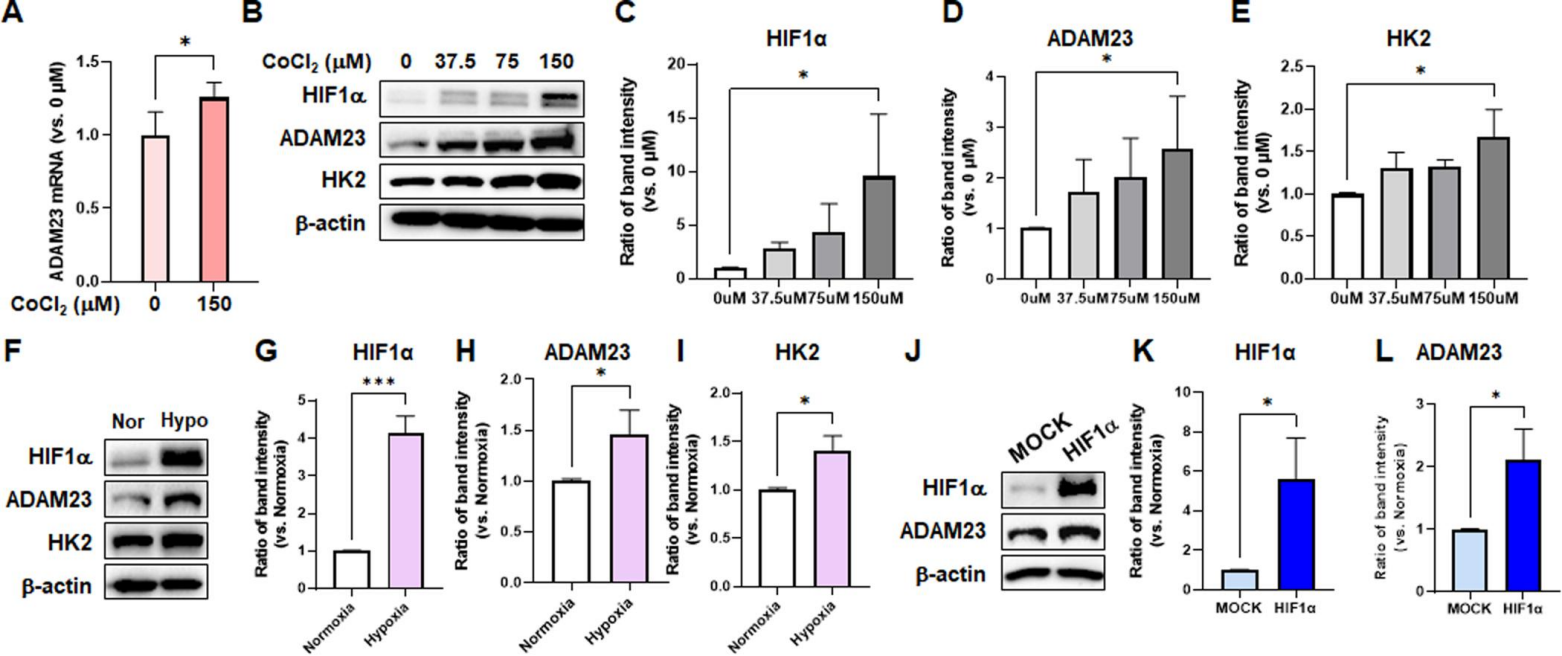
Upregulation of ADAM23 under hypoxic stress in Huh7 cells. (A) Quantitative RT–PCR analysis of ADAM23 mRNA expression in Huh7 cells treated with CoCl_2_ (150 µM, 24 h). (B) Immunoblot analysis of HIF1α, ADAM23, and HK2 in HepG2 cells following CoCl_2_ treatment (150 µM, 24 h); β‐actin served as a loading control. Full‐length blots are provided in Supporting Information S1: Figure 5A. (C–E) Densitometry intensity ratios for Figure 2B from replicated WB (*n* = 3). (F) Immunoblot analysis of HIF1α, ADAM23, and HK2 in Huh7 cells cultured under normoxic (21% O_2_) or hypoxic (1% O_2_) conditions for 24 h. Full‐length blots are provided in Supporting Information S1: Figure 6A. (G–I) Densitometry intensity ratios for Figure 2F from replicated WB (*n* = 3). (J) Immunoblot analysis of HIF1α, ADAM23, and HK2 in Huh7 cells following HIF1α overexpression. Full‐length blots in Supporting Information S1: Figure 7A. (K, L) Densitometry intensity ratios for Figure 2J from replicated WB (*n* = 3). Data are presented as mean ± SEM from three independent experiments. *p < 0.05, **p < 0.01, ***p < 0.001.

The hypoxic regions within tumors limit the availability of nutrients such as glucose [7]. To investigate whether glucose deprivation regulates ADAM23 expression, HepG2 and Huh7 cells were cultured under glucose‐deprived conditions. Glucose deprivation markedly increased ADAM23 expression in both HepG2 cells (Figure 3A,B) and Huh7 cells (Figure 3C,D). Hypoxic and glucose deprivation stresses are known to induce resistance to anti‐cancer drugs. To investigate this further, we established a sorafenib‐resistant HCC cell line using Huh7 cells (Huh7SR) [26]. We confirmed that these sorafenib‐resistant cells exhibited a significant upregulation of ADAM23 (Figure 3E,F). Since ADAM23 is a secretory protein, we collected the culture supernatants from Huh7 cells under both normoxic and hypoxic conditions, as well as from parental Huh7 and Huh7SR cells. Consistent with the intracellular protein expression patterns, hypoxic conditions significantly increased ADAM23 secretion compared to normoxia (Figure 3G,H). Furthermore, Huh7SR cells showed elevated levels of ADAM23 secretion compared to the parental Huh7 cells (Figures 3G,H). Altogether, these findings suggest that microenvironmental stresses, such as hypoxia and nutrient deprivation, induce the expression and secretion of ADAM23, which may contribute to the development of sorafenib resistance in HCC.

**Figure 3:**
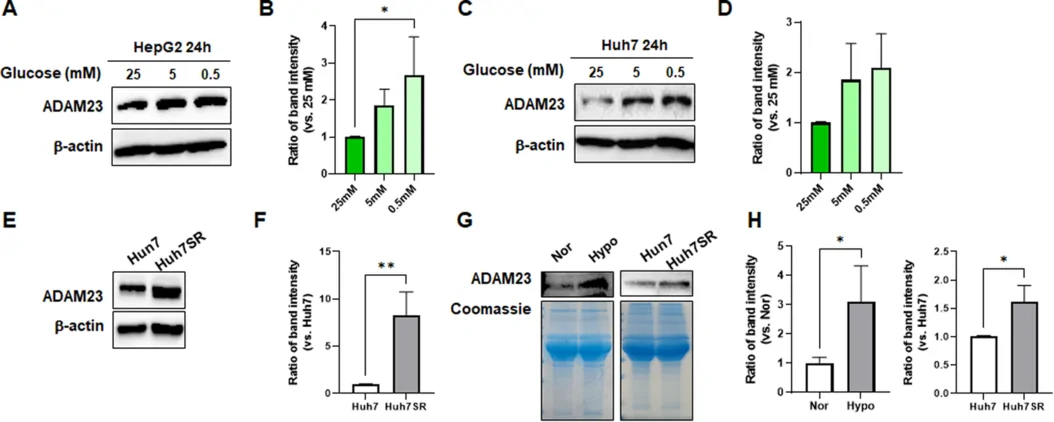
Metabolic stress and drug resistance induce ADAM23 expression and secretion. (A) Immunoblot analysis of ADAM23 expression in HepG2 cells cultured in media containing the indicated glucose concentrations for 24 h. β‐actin served as a loading control. Full‐length blots are provided in Supporting Information S1: Figure 8A. (B) Densitometry intensity ratios for Figure 3A from replicated WB (*n* = 3). (C) Immunoblot analysis of ADAM23 expression in Huh7 cells cultured in media containing the indicated glucose concentrations for 24 h. β‐actin served as a loading control. Full‐length blots are provided in Supporting Information S1: Figure 9A. (D) Densitometry intensity ratios for Figure 3C from replicated WB (*n* = 3). (E) Immunoblot analysis of ADAM23 in Huh7 and sorafenib‐resistant Huh7 (Huh7R) cells. β‐actin was used as a loading control. Full‐length blots are provided in Supporting Information S1: Figure 10A. (F) Densitometry intensity ratios for Figure 3E from replicated WB (*n* = 3). (G) Immunoblot analysis of ADAM23 expression in Huh7 cells under normoxic or hypoxic conditions (left), and in parental Huh7 versus sorafenib‐resistant Huh7R cells (right). Coomassie staining shows equal protein loading of supernatants. Full‐length blots are provided in Supporting Information S1: Figure 11A. (H) Densitometry intensity ratios for Figure 3G from replicated WB (*n* = 3).

#### 3.1.1 Effect of ADAM23 on Tumor Aggressiveness in HCC Cells and Patients

Glucose starvation and hypoxic stress have been reported to synergistically enhance the expression of epithelial–mesenchymal transition (EMT)‐related proteins [8]. To investigate whether ADAM23 contributes to EMT regulation, EMT‐associated protein expression was examined in ADAM23‐overexpressing HCC cells. E‐cadherin expression was decreased, whereas Snail and Slug levels were increased, suggesting that ADAM23 promotes the acquisition of mesenchymal characteristics in HCC cells (Figures 4A–4D). In line with the reduction in E‐cadherin expression, ADAM23‐overexpressing cells exhibited reduced epithelial clustering and a modestly more scattered morphology compared with control cells, although the overall cell morphology remained largely comparable (Supporting Information S1: Figure 1A). Together with the downregulation of E‐cadherin and upregulation of Snail and Slug, these findings suggest an early or partial EMT phenotype. Moreover, ADAM23 overexpression significantly increased hexokinase 2 (HK2) expression (Figure 4A,E), indicating enhanced glycolytic potential. Furthermore, ADAM23 overexpression resulted in a slight but significant increase in cell viability in both HepG2 cells (Figure 4F) and Huh7 cells (Supporting Information S1: Figure 1B). Collectively, these results suggest that elevated ADAM23 expression promotes mesenchymal phenotypes and enhances cellular viability in HCC cells.

**Figure 4:**
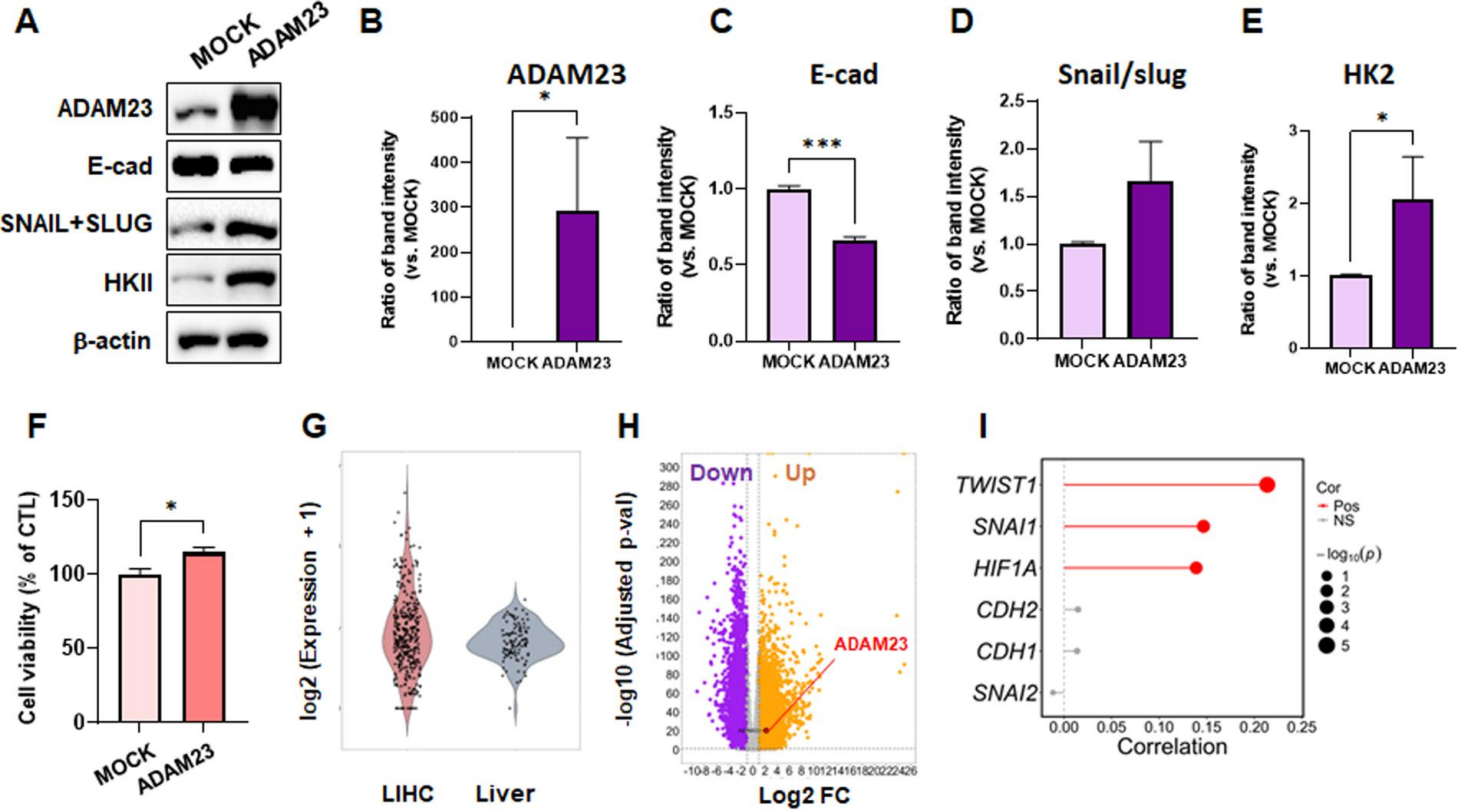
ADAM23 promotes tumor aggressiveness in HCC cells and correlates with hypoxia signaling and clinical outcomes. (A) Immunoblot analysis of ADAM23, E‐cadherin, Snail/Slug, and HK2 in HepG2 cells transfected with pCMV‐ADAM23 (1 µg) or mock vector for 48 h. β‐actin was used as a loading control. Full‐length blots are provided in Supporting Information S1: Figure 12A. (B–E) Densitometry intensity ratios for Figure 4A from replicated WB (*n* = 3). (F) Cell viability of HepG2 cells transfected with pCMV‐ADAM23 or mock vector for 48 h was assessed using the CCK‐8 assay. Data are presented as mean ± SEM. Statistical significance was determined using an unpaired two‐tailed Student's *t*‐test (*p* < 0.05). (G) ADAM23 mRNA expression in hepatocellular carcinoma (HCC) and normal liver tissues from the TCGA‐LIHC dataset (374 tumors vs. 49 normal samples). Data are shown as log_2_(expression + 1). (H) Volcano plot showing differential gene expression between HCC and normal liver tissues. (I) Lollipop plot illustrating the correlation of gene expression between ADAM23 and HIF1A, as well as EMT markers (CDH1, CDH2, SNAI1, SNAI2, VIM, and TWIST1) in the TCGA‐LIHC cohort.

To determine the clinical relevance of ADAM23, we analyzed its expression using the TCGA‐LIHC dataset. The mRNA expression of ADAM23 was significantly elevated in HCC tissues compared to normal liver tissues (log2 fold change = 2.209, FDR = 2.42 × 10^−21^), indicating a potential association with tumor progression (Figure 4G,H). Correlation analysis revealed significant positive correlations between *ADAM23* and *TWIST1*, *SNAI1*, and *HIF1A* (*p* < 0.05) (Figure 4I). Consistent with our results, *ADAM23* and *HIF1A* also showed a significant positive correlation in the TCGA dataset. Among EMT‐related markers, *TWIST1* exhibited the strongest correlations with *ADAM23*, followed by *SNAI1*, which was consistent with the protein expression pattern. In contrast, *CDH2* and *CDH1* showed no significant correlations with ADAM23, although a weak positive trend was observed.

To validate these findings in clinical specimens, immunohistochemical staining was performed using a tissue microarray containing 24 HCC lesions. ADAM23 expression showed a moderate positive correlation with HIF‐1α expression (*r* = 0.545; Figure 5A,B), supporting a potential link between ADAM23 expression and hypoxia‐related signaling. To evaluate the prognostic significance of ADAM23, patients were stratified into high‐ and low‐expression groups based on ADAM23 levels. Higher ADAM23 expression was associated with a trend toward reduced overall survival (OS) in the TCGA‐LIHC cohort (HR = 1.41, P = 5.84 × 10^−2^, Figure 5C). Taken together, these findings indicate that ADAM23 upregulation is associated with aggressive tumor characteristics and unfavorable clinical outcomes in HCC.

**Figure 5:**
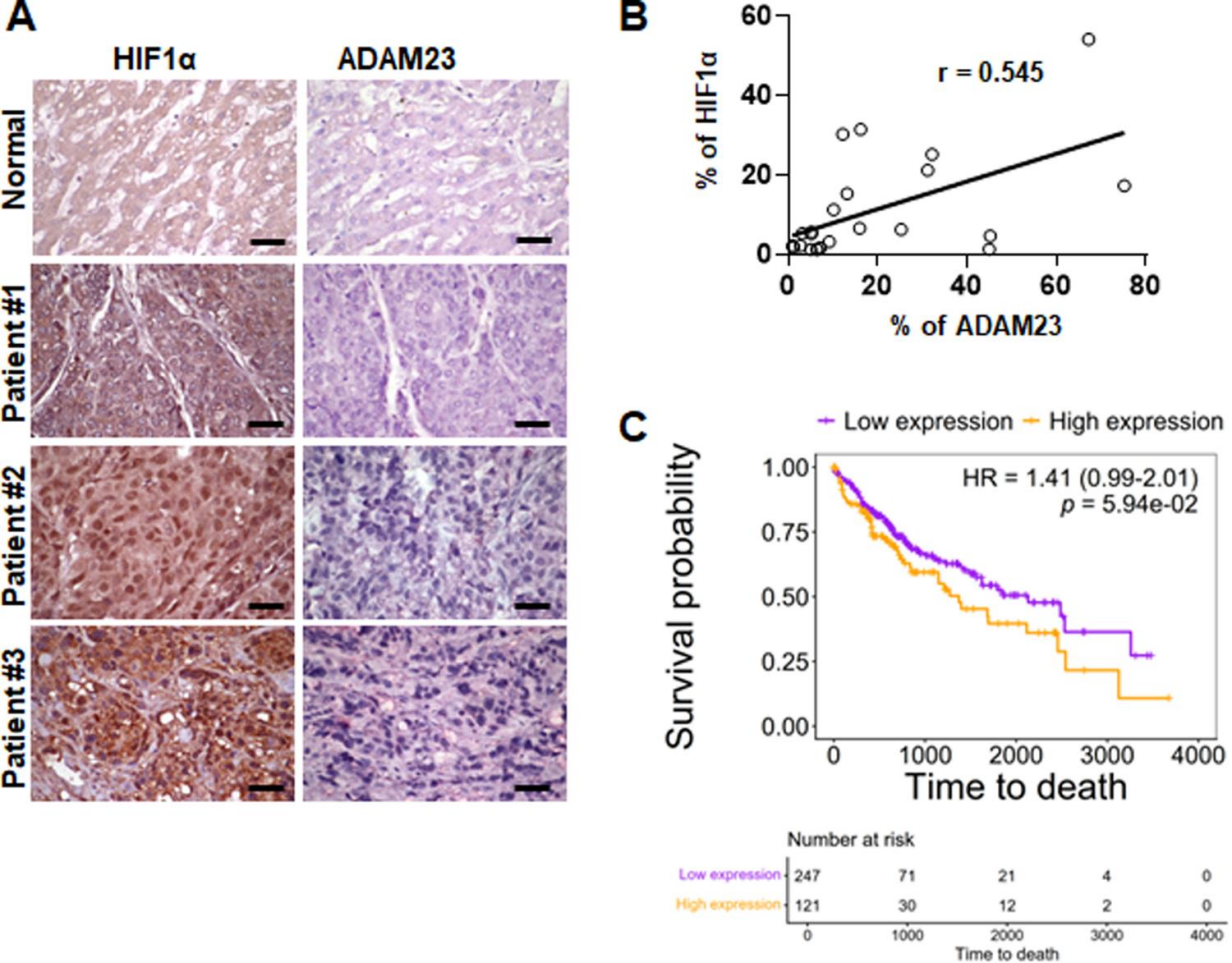
ADAM23 promotes tumor aggressiveness in patients with HCC. (A) Representative immunohistochemical co‐staining of ADAM23 and HIF‐1α in human HCC tissue microarrays (*n* = 24). ADAM23 is shown in magenta (AP substrate) and HIF‐1α in brown (HRP substrate). Scale bars, 50 µm. (B) Correlation between ADAM23‐positive and HIF‐1α‐positive areas in tumor regions from HCC specimens. Pearson's correlation coefficient was used. (C) Kaplan–Meier analysis of overall survival in HCC patients stratified by ADAM23 expression.

#### 3.1.2 Influence of ADAM23 on the Interplay Between HCC and Neuronal Cells

As ADAM23 has been reported to affect neuronal cells [14, 27], ADAM23 was overexpressed in SH‐Sy5Y neuroblastoma cells as a neuronal model. Overexpression of ADAM23 in SH‐SY5Y cells significantly, albeit modestly, increased cell viability and enhanced the expression of Ki‐67 and HK2, proliferation markers, while reducing the level of vimentin, a regulator of neuronal differentiation (Figure 6A–F). To examine whether ADAM23 derived from aggressive HCC cells influences neuronal cells that may, in turn, affect tumor cell behavior. Since ADAM23 expression was markedly higher in Huh7SR cells compared with parental Huh7 cells, conditioned media (CM) collected from Huh7 and Huh7R cells were subsequently applied to SH‐SY5Y neuronal cells (Figure 6G,H). Notably, ADAM23 levels were also elevated in the supernatant of Huh7SR‐CM relative to Huh7‐CM. Treatment of SH‐SY5Y cells with Huh7SR‐CM led to an increase in ADAM23 expression, accompanied by elevated levels of HK2 and Ki67, markers associated with cellular proliferation and metabolic activation (Figures 6G,I,J). These results indicate that ADAM23 contributes to communication between HCC and neuronal cells, promoting tumor progression through the establishment of a neuron–tumor communication axis.

**Figure 6:**
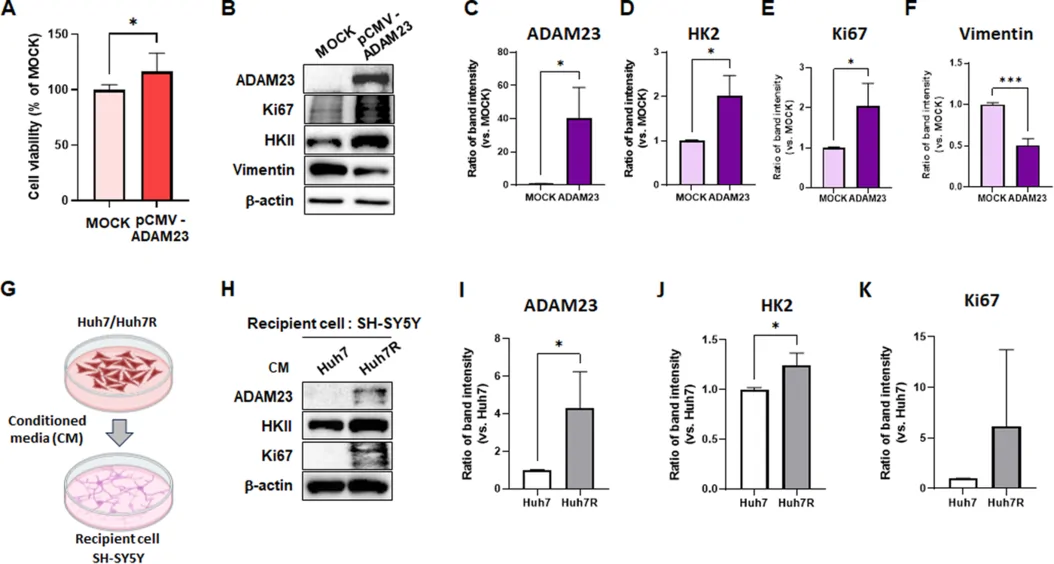
ADAM23 mediates reciprocal signaling between hepatocellular carcinoma and neuronal cells. (A) Cell viability of Sh‐Sy5Y cells transfected with 1 µg of pCMV‐ADAM23 or mock vector for 48 h was measured using the CCK‐8 assay. Statistical significance was determined using an unpaired two‐tailed Student's *t*‐test (*; *p* < 0.05). (B) Immunoblot analysis of ADAM23, Ki67, HK2, and vimentin expression in SH‐SY5Y cells transfected with 1 µg of pCMV‐ADAM23 or mock vector for 48 h. β‐actin was used as a loading control. Full‐length blots are provided in Supporting Information S1: Figure 13A. (C–D) Densitometry intensity ratios for Figure 4A from replicated WB (*n* = 3). (G) Immunoblot analysis of ADAM23, HK2, and Ki‐67 expression in SH‐SY5Y cells following incubation with conditioned media (CM) derived from Huh7 or sorafenib‐resistant Huh7SR cells for 48 h. β‐actin served as a loading control. Full‐length blots are provided in Supporting Information S1: Figure 14A. (H–J) Densitometric intensity ratios for Figure 4A from replicated WB (*n* = 3).

#### 3.1.3 Co‐Culture of Huh7 and SH‐SY5Y Cells

To further evaluate the reciprocal interaction between HCC and neuronal cells, we established a co‐culture spheroid model using GFP‐tagged SH‐SY5Y cells with either Huh7 or Huh7SR cells. After 6 days of co‐culture, spheroids formed by Huh7SR and SH‐SY5Y cells were noticeably larger than those formed by Huh7 and SH‐SY5Y cells. In addition, GFP fluorescence intensity was significantly increased in Huh7SR/SH‐SY5Y spheroids, suggesting enhanced SH‐SY5Y cell expansion in the presence of sorafenib‐resistant HCC cells (Figure 7A,C).

**Figure 7:**
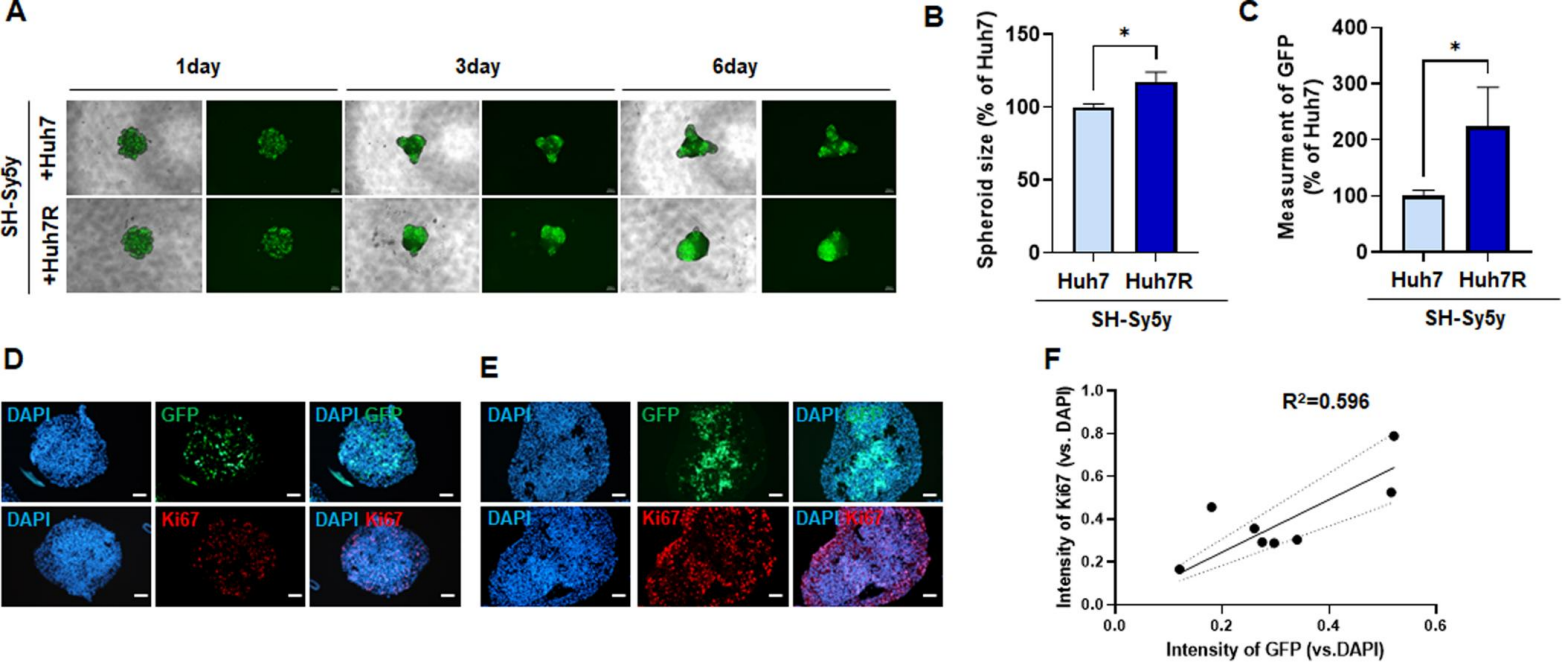
Co‐culture spheroid model reveals enhanced neuronal proliferation in the presence of sorafenib‐resistant HCC cells. (A) Representative bright‐field and fluorescence images of co‐culture spheroids formed by GFP‐labeled SH‐SY5Y cells with either Huh7 or sorafenib‐resistant Huh7R cells after 6 days. Scale bars, 200 µm. (B) Quantification of spheroid diameter showing increased size in Huh7SR + SH‐SY5Y spheroids compared with Huh7 + SH‐SY5Y spheroids. (C) Quantification of GFP fluorescence intensity indicating enhanced neuronal cell proliferation and/or survival in co‐cultures with Huh7SR cells. (D) Representative fluorescence images showing Ki67 staining of co‐culture spheroids formed by GFP‐labeled SH‐SY5Y cells with either Huh7 or sorafenib‐resistant Huh7R cells after 6 days. Scale bars, 100 µm. (F) Scatter plot showing the relationship between GFP intensity (normalized to DAPI) and Ki67 intensity (normalized to DAPI). Each dot represents an individual spheroid. The solid line indicates the linear regression fit, and the dotted lines represent the 95% confidence interval. Data represent mean ± SEM from three independent experiments. Statistical significance was determined using an unpaired two‐tailed Student's *t*‐test (*p* < 0.05; *p < 0.01; **p < 0.001).

Ki‐67 staining was subsequently performed to assess proliferative activity within the spheroids. Quantitative analysis revealed a positive correlation between GFP and Ki‐67 intensities across spheroids (*R* ^2^ = 0.596; Figure 7D–F), indicating that spheroids containing more SH‐SY5Y cells exhibited higher overall proliferative activity. Due to technical limitations of the 3D immunofluorescence analysis, Ki‐67 expression specifically within GFP‐positive SH‐SY5Y cells could not be directly determined. Nevertheless, these findings suggest that reciprocal interactions between HCC and neuronal cells contribute to enhanced proliferative activity within the 3D tumor microenvironment.

## 4 Discussion

Hypoxia is a main driver of tumor progression and therapeutic resistance in HCC [7]. Consistent with previous studies showing that HIF1α orchestrates the transcription of genes involved in glycolysis, angiogenesis, and epithelial–mesenchymal transition (EMT) [7, 28, 29], our study identified ADAM23 as a novel hypoxia‐related gene/protein in HCC cells. Under both CoCl_2_‐induced and low‐oxygen conditions, ADAM23 expression was upregulated along with HK2, classical markers of metabolic reprogramming.

Although ADAM23 has been characterized as a tumor suppressor in several malignancies, including breast, lung, and brain cancers [30], Costa et al. demonstrated that, in heterotypic tumor environments, ADAM23‐negative cells can paradoxically promote tumor growth and metastasis by stimulating the proliferation and invasion of neighboring ADAM23‐positive cells through the secretion of LGI4 and nitric oxide (NO) [31]. These findings suggest that intercellular communication and the tumor microenvironment determine whether ADAM23 functions as a tumor suppressor or oncogene. Consistent with this concept, our data further show that ADAM23 expression is induced under hypoxic and glucose‐deprived conditions, two prominent stressors within the HCC microenvironment. The induction of ADAM23 under these distinct conditions suggests that its expression is responsive to diverse microenvironmental cues rather than being controlled by a single regulatory pathway. Hypoxic induction of ADAM23 was associated with HIF1α signaling, whereas glucose deprivation activates the PERK–eIF2α–ATF4 axis of the integrated stress response [32], which regulates selective stress‐responsive transcriptional programs. Collectively, these findings suggest that, at least in HCC cells, ADAM23 acts as a suppressor under normal conditions but shifts to a tumor‐promoting role under hypoxic conditions, including glucose deprivation.

Our study also highlights the potential role of ADAM23 in mediating communication between HCC cells and neuronal cells of the tumor microenvironment. Increasing evidence indicates that neuron–tumor crosstalk contributes to cancer progression in several malignancies, including pancreatic and prostate cancers, where neural infiltration enhances tumor growth and dissemination [33, 34, 35, 36, 37, 38]. In this context, we observed that sorafenib‐resistant Huh7SR cells expressed markedly higher levels of ADAM23 than parental Huh7 cells. Conditioned media from Huh7SR cells induced the expression of ADAM23, Ki67, and HK2 in neuronal SH‐SY5Y cells. These findings suggest that ADAM23 may function as a molecular mediator of neuron–tumor communication in the liver, contributing to the establishment of a supportive neural niche that facilitates tumor adaptation under hypoxic and drug‐resistant conditions.

Our co‐culture spheroid experiments further support our findings in 2D culture. Spheroids composed of sorafenib‐resistant Huh7SR and SH‐SY5Y cells were larger and exhibited stronger GFP fluorescence than those formed with parental Huh7 cells, suggesting enhanced tumor cell and neuronal cell proliferation or survival. Previous reports determined that tumor‐secreted factors such as NGF or BDNF can drive neuronal remodeling and proliferation in pancreatic and gastric cancers [39, 40, 41]. Unlike these studies, which primarily focused on neurotrophic factors, our data implicate a membrane‐associated metalloproteinase, ADAM23, as a key component of cross‐linker between HCC and neuron cells. ADAM23 derived from HCC827SR cells appeared to influence the differentiation of neuronal cells, as evidenced by a decrease in vimentin expression. This observation suggests that ADAM23 promotes neuronal differentiation, consistent with the role of vimentin as a marker of undifferentiated or immature neural cells. Previous studies have reported that neuronal differentiation within tumors can affect tumorigenesis, supporting our findings [42, 43, 44].

Clinically, analysis of the TCGA cohort revealed that ADAM23 expression was significantly elevated in HCC tissues and positively correlated with HIF1α expression, consistent with our experimental data linking ADAM23 induction to hypoxia. These results align with previous reports showing that high HIF1α activity predicts poor prognosis and therapeutic resistance in HCC [45, 46]. Notably, a recent study developed a cholinergic neuronal score in sorafenib‐treated HCC patients and demonstrated that higher cholinergic signatures were associated with more aggressive tumor phenotypes and poorer therapeutic outcomes [47]. However, in this study, the precise mechanism by which ADAM23 mediates reciprocal signaling between tumor and neuronal cells remains incompletely understood. Whether this interaction involves direct cell‐to‐cell contact, extracellular vesicles, or proteolytic activation of downstream substrates requires further investigation. Comprehensive omics‐based analyses following ADAM23 modulation could help identify its downstream effectors and signaling partners. Although this study could not fully elucidate the underlying mechanism, the clinical observations together with our experimental data suggest that ADAM23 may act as a key mediator connecting hypoxic adaptation with neuron–tumor communication, thereby contributing to the aggressive and drug‐resistant phenotype of HCC.

Recent studies have identified immature, cholinergic‐oriented intratumoral neural populations in human HCC [47]. In this study, we used SH‐SY5Y cells as a neuronal model; however, these cells represent only a partial surrogate for the heterogeneous autonomic innervation of the liver. SH‐SY5Y cells contain interconvertible neuroblastic (N‐type) and substrate‐adherent (S‐type) populations [48], and vimentin, a mesenchymal marker enriched in S‐type cells, is downregulated during neuronal differentiation [49]. Therefore, the reduced vimentin expression observed after ADAM23 overexpression is consistent with a shift toward a more neuronal phenotype, although it does not establish acquisition of a specific neurotransmitter identity. Further studies using cholinergic markers, such as ChAT and VAChT, and more physiologically relevant neuronal models will be required to define the identity and function of HCC‐associated neurons [47]. In addition, while our findings identify ADAM23 as a regulator of a supportive neural niche, the downstream neurotransmitters and signaling mediators responsible for HCC chemoresistance remain to be determined.

In conclusion, our study identified ADAM23 as a hypoxia‐responsive factor that links metabolic stress to neuron–tumor communication in HCC cells. Through experimental and clinical analyses, we demonstrated that ADAM23 expression is induced under hypoxic and glucose‐deprived conditions and is associated with aggressive, drug‐resistant tumor phenotypes. Moreover, ADAM23 appeared to influence the growth and differentiation of neuronal cells within the tumor microenvironment. These findings suggest that ADAM23 may act as a mediator connecting hypoxic adaptation with neural remodeling in tumors. Targeting the ADAM23‐mediated signaling axis may therefore represent a promising therapeutic strategy to overcome drug resistance and suppress tumor progression in HCC especially under hypoxia or nutrient‐deprived conditions.

## Author Contributions

T. Park, S. Kim, and H. Cho performed the experiments, analyzed the data, and wrote and revised the manuscript. J. Nam performed the statistical and bioinformatics analyses and assisted with data interpretation. D. Choi contributed to data interpretation and revised the manuscript. M. Lee conceived and designed the study, supervised the project, and wrote and revised the manuscript. All authors have read and approved the final version of the manuscript.

## Ethics Statement

The authors have nothing to report.

## Consent

The authors have nothing to report.

## Conflicts of Interest

The authors declare no conflicts of interest.

## Supporting information


Supporting File

## Data Availability

The data that support the findings of this study are available from the corresponding author upon reasonable request. All data generated or analyzed during this study are included in this published article and its supplementary information files.
