# Supplementary material for: Hypoxia‐Induced ADAM23 Drives Neuron–Tumor Crosstalk and Therapeutic Resistance in Hepatocellular Carcinoma
Source: Mol Carcinog. 2026 Sep 3;65(12):1533–43. doi: 10.1002/mc.70174 (PMC13757747; doi:10.1002/mc.70174)
Supplement: Supplementary file 1 [file MC-65-1533-s001.docx]

**Supplementary Fig1.** (A) Phase-contrast images of MOCK- and ADAM23-transfected HepG2 cells. HepG2 cells were transfected with either the ADAM23 expression vector or the MOCK vector. Representative phase-contrast images of cell morphology are shown. Scale bars, 50 μm. (B) Cell viability of Huh7 cells transfected with ADAM23 or mock vector. Cell viability of Huh7 cells transfected with ADAM23 or mock vector.for 48 h was assessed using the CCK-8 assay. Data are presented as mean ± SEM. Statistical significance was determined using an unpaired two-tailed Student’s t-test (p < 0.05).

**Supplementary Fig 2.** (A) Whole blot images of Fig 1B.

**Supplementary Fig 3.** (A) Whole blot images of Fig 1F.

**Supplementary Fig4.** (A) Whole blot images of Fig 1J.

**Supplementary Fig5.** (A) Whole blot images of Fig 2B.

**Supplementary Fig 6.** (A) Whole blot images of Fig 2F.

**Supplementary Fig7.** (A) Whole blot images of Fig 2J.

**Supplementary Fig8.** (A) Whole blot images of Fig 3A.

**Supplementary Fig9.** (A) Whole blot images of Fig 3C.

**Supplementary Fig10.** (A) Whole blot images of Fig 3E.

**Supplementary Fig11.** (A) Whole blot images of Fig 3G.

**Supplementary Fig12.** (A) Whole blot images of Fig 4A.

**Supplementary Fig13.** (A) Whole blot images of Fig 6B.

**Supplementary Fig14.** (A) Whole blot images of Fig 6B.
